# HappyMums mobile application study protocol: use of a smartphone application to gather data predictive of antenatal depression

**DOI:** 10.1136/bmjopen-2025-106978

**Published:** 2026-02-04

**Authors:** Kristi Priestley, Riddhi Laijawala, Katie Hazelgrove, Rebecca Bind, Lavinia Rebecchini, Nicole Mariani, Sorcha Alford, Madeline Kirkpatrick, Francesca Mancino, Seungyoung Kim, Suvasthiga Pushpakanthan, Alessandra Biaggi, Libera Cavaliere, Maria Grazia Di Benedetto, Marijana Matijaš, Maja Žutić, Maja Brekalo, Sandra Nakić Radoš, Katarzyna Żukowska, Anna Braniecka, Marta Jackowska, Margherita Bessi, Elena Agnoletto, Elisa Maria Teresa Melloni, Francesco Benedetti, Maria Bulgheroni, Margherita La Gamba, Carlos Martín Isla, Cristian Izquierdo Morcillo, Karim Lekadir, Verna Salo, Tiina Seikku, Katri Räikkönen, Malvika Godara, Ulrike Maria Schneider-Schmid, Sonja Entringer, Claudia Buß, Deirdre de Barra, Anthony Woods, Paola Dazzan, Annamaria Cattaneo, Carmine Pariante

**Affiliations:** 1 Department of Psychological Medicine, King’s College London Institute of Psychiatry Psychology and Neuroscience, London, England, UK; 2 Department of Pharmacological and Biomolecular Sciences, University of Milan, Milan, Italy; 3 Biological Psychiatry Unit, IRCCS Istituto Centro San Giovanni di Dio Fatebenefratelli, Brescia, Italy; 4 Department of Psychology, Catholic University of Croatia, Zagreb, Croatia; 5 Institute of Psychology, SWPS University, Warsaw, Poland; 6 Faculty of Psychology, SWPS University, Warsaw, Poland; 7 Psychiatry and Clinical Psychobiology Unit, Division of Neurosciences, IRCCS San Raffaele Scientific Institute, Milan, Italy; 8 R&D, Ab.Acus srl, Milan, Italy; 9 Barcelona Artificial Intelligence in Medicine Lab (BCN-AIM), Facultat de Matemàtiques i Informàtica, Universitat de Barcelona, Barcelona, Spain; 10 Institució Catalana de Recerca i Estudis Avançats (ICREA), Barcelona, Spain; 11 Department of Psychology, University of Helsinki Finland, Helsinki, Finland; 12 Institute of Medical Psychology, Charité—Universitätsmedizin Berlin, Corporate Member of Freie Universität Berlin and Humboldt-Universität zu Berlin, Institute of Medical Psychology, Berlin, Germany; 13 German Center for Child and Adolescent Health, Berlin, Germany; 14 Development, Health, and Disease Research Program, University of California, Irvine, California, USA; 15 German Center for Mental Health, Berlin, Germany; 16 Tommy’s, United Kingdom, London, UK; 17 Department of Psychosis Studies, Institute of Psychiatry, Psychology and Neuroscience, King’s College London, London, UK

**Keywords:** Pregnancy, Protocols & guidelines, Mobile Applications

## Abstract

**Introduction:**

Mobile health (mHealth) technologies have become increasingly popular for monitoring mental health symptoms and lifestyle behaviours, and are largely reported to be feasible and acceptable to users. However, to date, the efficacy of such technologies to improve perinatal mental health outcomes has been mixed. Within the perinatal context, much of this work has been done in the context of postpartum depression, stemming from electronic health records as well as cohort studies. There is, however, a dearth of studies focusing on depression in pregnancy, and machine learning-based clinical decision support systems remain underexplored. The HappyMums application has been developed to meet this need, and its use across Europe will be tested in this study.

**Methods and analysis:**

A total of 1000 pregnant people currently suffering from, or at risk of, antenatal depression will be recruited across six countries. All participants will be between 13 and 28 weeks’ gestation and will be given access to the new purposefully developed HappyMums mobile application, to use from enrolment until 2 months postpartum. The application leverages passively collected data from smartphone sensors relating to physical activity and behaviour, as well as requiring active engagement from the user to complete mental health questionnaires and ‘game-like’ activities. Digital data types will be combined with traditional mental health measurement methods, such as standardised questionnaires and interviews, to develop novel predictive models capable of identifying mental health trajectories in women at risk of developing antenatal depression and to test the app’s utility for use as personalised risk prediction and depression identification tool. The primary outcome of this study is to determine what proportion of users will continue to use the mobile application and engage with its tasks and activities at least weekly, while secondary exploratory outcomes include assessing usability of the app and testing the predictive ability of a novel machine learning-based model. These outcomes will, for the first time, be assessed by integrating active as well as passive data.

**Ethics and dissemination:**

Ethical approval has been granted by local research ethics committees in each recruiting centre. At King’s College London (leading the clinical study), the study was reviewed by the East of England—Essex Research Ethics Committee and granted favourable opinion (REC reference 24/EE/0129). All other sites collecting participant data have the study approved for local delivery. Findings relating to the primary and secondary outcomes will be submitted for publication in open access, peer-reviewed journals, as well as presentations at conferences as symposia or posters. Findings will be made available to a non-specialist audience through open access digital mental health magazines and promotion on social media.

**Trial registration number:**

NCT06578845.

**STRENGTHS AND LIMITATIONS OF THIS STUDY**

- This is a Europe-wide study that will recruit pregnant people between 13 and 28 weeks’ gestation, to use a purposefully-developed mobile application in order to better understand depression in pregnancy and its associated risk factors.
- The application will leverage active as well as passive data collection to build models capable of predicting antenatal mental health trajectories that are relevant for clinicians working with perinatal populations.
- The study will provide insight into the ever-expanding digital mental health space, with a focus on antenatal mental health, which is often overlooked when developing biomedical technology.
- A current limitation of this methodology is that it excludes individuals who lack access to an appropriate smartphone, who may be part of a deprived demographic group and experience many barriers to care.
- Additionally, we recognise that methodological limitations include heterogeneity in data collected (due to different cultural contexts), anticipated technical challenges, concerns about data sharing and potential burden on participants in filling out self-report questionnaires. These limitations have been identified and mitigation strategies have been implemented.

## Introduction

 The global prevalence of antenatal depression (AD) is estimated to range from 15% to 65%,^1^ with commonly-reported risk factors including history of depression, low social support, the occurrence of major life events and history of abuse.^2^ AD can have a detrimental impact not just on the mother herself, but also on her offspring. It is therefore vital to further develop our understanding of its risk factors and early warning signs, in order that we can aid early identification and treatment of AD. This will in turn allow us to minimise risk and support well-being for both the mother and her child.

Furthermore, screening interventions have been shown to be cost-effective^3 4^ and they have been strongly recommended by recent evidence-based clinical practice guidelines for peripartum depression.^5^

Digital mental health is a developing field in which technological advancements are being harnessed to support mental well-being and reduce resource requirements, so that support can be provided to those who most need it. These include technologies to improve healthcare processes and pathways, for example, technologies implemented in healthcare settings to facilitate communication between healthcare providers, with some evidence that this can reduce patient wait time.^6^ The accessibility of these technologies gives them particular promise for improving access to care in high-need communities or demographic groups who are unable or unwilling to engage fully with traditional healthcare services due to numerous barriers in place.

This technology is also being used in patient care and management. The developing field of mobile health (mHealth) applications includes the use of devices and software to provide psychological and behavioural interventions, as well as systems designed to identify and monitor changes in symptoms.^7^ mHealth services are generally reported to be positive for users due to their flexibility and accessibility.^8^ There also is some evidence of beneficial effects, for example, one randomised trial investigating a ‘mood tracking and alert’ application, which provides information to care providers and alerts them when participant mood symptoms worsen, demonstrated improved depression and anxiety scores in users over the 8 weeks of use.^9^

In the perinatal period, perhaps due to the heterogeneity of experience in this time of great change, findings are mixed with regards to the effectiveness of using such mobile applications to promote better physical health in pregnancy, with one meta-analysis reporting positive efficacy,^10^ but another review reporting that there is limited evidence to draw firm conclusions on their effectiveness to improve pregnancy health and behaviour change.^11^

Focusing specifically on mental health, a scoping review of available mHealth applications for perinatal depression and anxiety identified 22 unique studies,^12^ of which 12 were mHealth and 10 were texting-based interventions, including interventions such as psychoeducation, peer support and psychological therapy. The efficacy of these interventions was mixed, but generally they showed positive impacts on depressive symptoms and some psychosocial outcomes.^13^ Other smartphone-based interventions, such as mindfulness, have also shown some efficacy in improving perinatal depressive symptoms,^14 15^ as well as improving associated sequelae such as birth outcomes.

Taking this one step further, mHealth technologies have been explored from the perspective of building machine learning models to aid clinical decision-making.^16^ Indeed, evidence from scoping reviews has identified that passive data such as step count, light exposure and speech patterns have been able to predict symptom severity among adult populations with depression.^17^ Within the perinatal context, much of this work has been done in the context of postpartum depression, stemming from electronic health records^18^ as well as cohort studies.^19^ There is, however, a dearth of studies focusing on depression in pregnancy, and machine learning-based clinical decision support systems remain underexplored.^20^ Mobile applications collecting self-reported clinical and socio-demographic data have been able to produce highly predictable models of depression in pregnancy.^21^ Among these, studies integrating passive sensing have been explored in the context of maternal depression, finding that Global Positioning System (GPS) data has indeed been associated with depressive symptomology.^22^ However, studies looking at integrating passive as well as active data, especially among perinatal populations, are limited.

Given the important links between physical, behavioural and mental health, there may be additional benefit to monitoring behaviours such as movement and activity alongside perinatal mental health. Indeed, a mobile application designed to monitor daily movement patterns in pregnant women at risk for perinatal depression found that milder depression was associated with a larger daily travel radius, and worsening mood was associated with a contracted radius of travel, while no such associations were found with anxiety.^16^

However, bringing together evidence on currently available mHealth apps, a recent systematic review and meta-analysis concluded they do not appear to consistently and effectively reduce symptoms of perinatal depressive symptoms. The authors noted a need for the development of higher quality apps in order to improve outcomes.^18^ Despite these challenges in efficacy, evidence still suggests that patients in antenatal care find these methods acceptable—a feasibility study of administering mood screening questionnaires and Cognitive Behavioral Therapy (CBT)-based information by text, to women in antenatal care, reported that 92% found it easy to receive the texts, 72% found it easy to complete the questionnaires and 76% provided overall positive feedback.^19 20^

Given the apparent willingness of antenatal populations to engage with these technologies, and the fact that some studies report efficacy, we hypothesise that the quality of apps, and therefore their effectiveness, could be improved if an app were developed to combine many of the functionalities previously reported—including symptom monitoring, psychoeducation resources for perinatal mental health and passive data collection, as well as reporting of findings to clinicians in a timely way. Indeed, a qualitative study of using apps for psychological self-reporting identified the importance of providing value to users through both self-report and supplementary features to allow for longitudinal user engagement.^21^

As such, to develop knowledge and understanding of the use of mobile technology for antenatal depressive symptom monitoring, the HappyMums App Study was designed to develop and evaluate a new app that will combine the multiple methodologies identified in the literature as having putative efficacy in perinatal depression: active symptom monitoring through smartphone-based questionnaires, monitoring of behavioural and activity changes through passive data collection and the provision of supplementary features of benefit during pregnancy, to support longitudinal engagement. Additionally, the HappyMums app will be made available to pregnant users across 6 European countries (Croatia, Finland, Germany, Italy, Poland and the UK) to investigate its use in various cultural contexts. Machine learning techniques will then be used following completion of data collection to aggregate multiple data types, and the resulting models will be tested for their capabilities of predicting and identifying AD, as well as response to treatment.

### Aims

1. To test the new HappyMums app, which will include smartphone-based questionnaires, passive data collection, game-style emotional recognition and cognitive tests and psychoeducational material.
2. To determine whether pregnant people, at risk of, or currently suffering from AD, find it acceptable and feasible to use the ‘HappyMums’ mobile application through trimesters two and three of pregnancy, as a method of passively and actively collecting data which could be used to monitor their mental health during pregnancy.
3. To use the data collected by the mobile application to build models capable of predicting antenatal mental health trajectories, using ‘traditional’ approaches such as standardised questionnaires and interview as comparators.

### Objectives and outcomes

To address these aims, we have developed this protocol, with the following objectives and outcomes.

#### Primary objective

Do pregnant people feel able to engage with the ‘HappyMums’ mobile application from their point of enrolment in the study, until the end of their pregnancy?

Outcome: the primary outcome of this study is to determine what proportion of users will continue to use the mobile application and engage with its tasks and activities at least weekly from the point at which they are given access to it, until the end of their pregnancy.

#### Secondary objectives

1. To assess the usability of the application, the ‘Systems Usability Scale’ and ‘Unified Theory of Acceptance and Use of Technology’ questionnaires will be administered to users after they cease their use of the application (after giving birth).
2. To test the predictive ability of the model, using data passively and actively collected by the application, prediction of antenatal mental health trajectories of users as determined by the app will be compared with standardised measures via the Edinburgh Postnatal Depression Scale (EPDS) and the Patient Health Questionnaire 9 item, available for app users to complete every 2 weeks. A separate subanalysis will be conducted in the subsample receiving a comprehensive, face-to-face clinical assessment with the Mini International Neuropsychiatric Interview (MINI) at baseline and in the early postpartum.

#### Exploratory objectives

1. To test the association between physiological biomarkers (obtained from blood and saliva samples collected during pregnancy and the early postpartum) and digital markers collected via the mobile application.
2. To test the association between measures of mother–infant interaction in the early postpartum using the Crittenden Child Adult Relationship Experimental Index (CARE-Index)^23^ and digital markers collected via the mobile application, in the subsample receiving a comprehensive, face-to-face clinical assessment, including a video to assess this.

## Methods and analysis

### Data collection plan

Data will be collected according to the data collection timeline in table 1.

**Table 1:** Timeline of measures

Time point	In app	By researchers
Baseline/enrolment		Demographics, EPDS, PHQ-9, GAD-7, CAS, AAQ
		(Comprehensive/validation subsample only: CSI, MINI, CTQ)
Week 25	Cognitive tasks	(Comprehensive/validation subsample only: MSPSS, MAAS, PSS)
Every 2 weeks	EPDS, PHQ-9, GAD-7	
Week 27	Emotion recognition tasks	
Week 35	Cognitive tasks	(Comprehensive/ validation subsample only: MSPSS, MAAS, PSS)
Week 37	Emotion recognition tasks	
From birth to 2 months postpartum	PHQ-9, GAD-7, EPDS	Birth questionnaire, BLE, SUS, UTAUT (Comprehensive/ validation subsample only: PBQ, CSI, MINI)

AAQ, Adult Attachment Questionnaire; BLE, Brief Life Events; CAS, Composite Abuse Scale; CSI, Couple Satisfaction Index; CTQ, Childhood Trauma Questionnaire; EPDS, Edinburgh Postnatal Depression Scale; GAD, Generalised Anxiety Disorder Assessment; MAAS, Maternal Antenatal Attachment Scale; MINI, Mini International Neuropsychiatric Interview; MSPSS, Multidimensional Scale of Perceived Social Support; PHQ, Patient Health Questionnaire; PSS, Perceived Stress Scale; SUS, Systems Usability Scale; UTAUT, Unified Theory OF Acceptance and Use of Technology.

### Study design

The HappyMums mobile application study is comprised of seven similar studies being conducted at seven recruitment sites across Europe and coordinated by King’s College London (UK). The other six sites are: University of Milan (Italy), Ospedale San Raffaele (Italy), Charité (Germany), University of Helsinki (Finland), SWPS University (Poland) and Catholic University of Croatia (Croatia). This work is part of the HappyMums Consortium, explained here,^24^ funded by the European Commission. All sites will follow the protocol as described here and aggregate data following data collection completion. The study commenced on 01 December 2022 with weekly co-creation meetings to develop the study protocol and will end on 31 October 2026.

### Study flow chart

**Figure 1:**
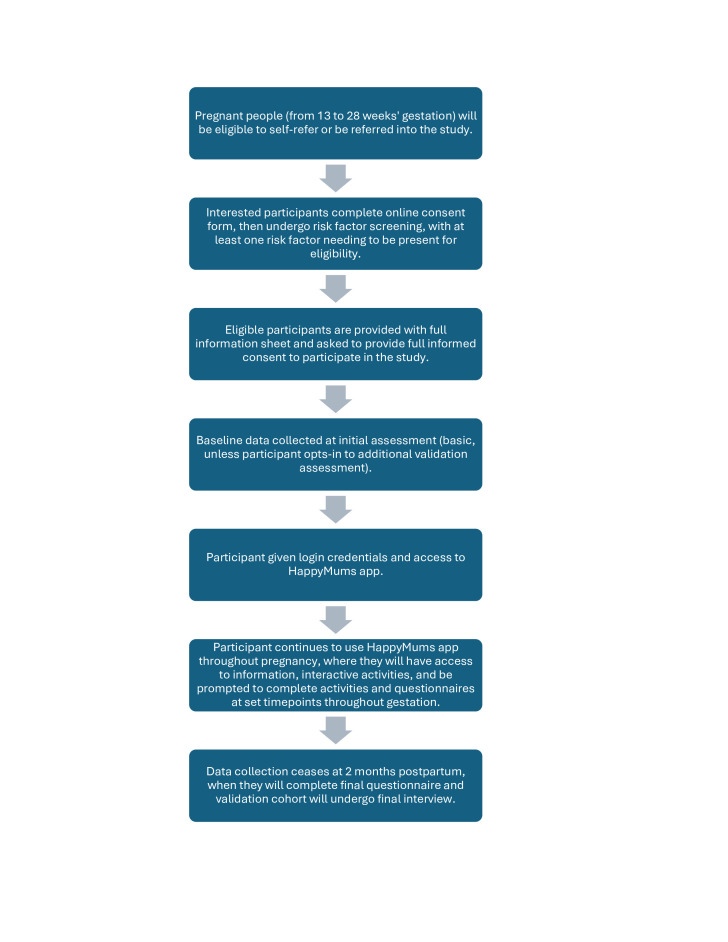
HappyMums study flowchart.

Figure 1 shows a flowchart summarising the recruitment, enrolment and application usage timelines for the study.

### Participants

Participants for this study will be n=1000 pregnant people (approximately 150 per centre), who are either currently suffering with AD symptoms, or meet the criteria for at least one risk factor for AD, as determined by the expertise of the consortium, literature searches and the current availability of screening questionnaires. Eligibility will be determined using a screening questionnaire specifically created within the consortium, as shown in online supplemental material.

Inclusion criteria: pregnant participants, aged 18 or older, enrolled from 13 to 28 weeks’ gestation and scoring >0 on the screening instrument. Participants must have a satisfactory understanding of the national language of their recruitment site, in order to provide fully informed consent. They must also own a smartphone capable of downloading and running the HappyMums application, which will be available in all national languages.

Exclusion criteria: Inability to give informed consent.

### Sample size

Given that this study is conducting exploratory analyses regarding machine-learning-generated predictive values of application-derived datasets, a power calculation was not deemed appropriate. However, instead, the consortium determined approximately 150 participants per recruitment site (1000 in total) to be a feasible recruitment target within the timescales allowed by the consortium work plan, while being large enough to represent the cultural and demographic differences across contexts.

### Recruitment process

Prospective participants will be recruited via different pathways, such as advertisements on social media and other online spaces for women in the perinatal period, as well as posters and flyers in spaces relevant to pregnant women, such as antenatal clinics, doctors’ surgeries, children and family centres and relevant newsletters. Other recruitment pathways include referrals from departments of obstetrics and gynaecology at hospitals, with appropriate healthcare professionals referring suitable participants for the study. Prospective participants will be informed about the study procedure and will provide full written consent before entering the study.

Participants will be asked to complete the screening questionnaire. After completion, they will be contacted by researchers from their host centre to discuss the outcome of the screening and their eligibility for the study. Eligible participants will undergo an initial assessment before being given access to the mobile application.

Of note, it was deemed important by consortium partners that at least a subset of participants undergo a clinical interview to determine psychiatric status before and during the study, in order to assess the mobile application’s validity. However, given the length of the interview may be prohibitive for some participants, and therefore, to increase inclusivity, it was decided that a clinical interview would not be mandatory for all participants, but instead that there would be an opt-in ‘comprehensive’ cohort of at least 30 participants per site, in which willing participants could volunteer to undergo clinical interview the MINI^25^ at baseline and post birth. They will also be asked to complete additional measures to determine risk factor prevalence and implications for antenatal mental health (more information below and in table 1).

### The mobile application

The HappyMums mobile application, developed by Ab.Acus (Italy), is a novel digital platform, designed for use by pregnant people, for risk detection, intervention and symptom monitoring. The overall aim is to develop a data collection device which could in future be paired with a dashboard for the patient’s clinician to view their data for use in clinical and treatment decisions. Ab.Acus worked closely with experts in the field of perinatal psychiatry within the consortium to develop the app.

For the purposes of this study, this dashboard will be made available to researchers and clinicians associated with the study in order to monitor the data entry of study participants; however, it will not be made available to the participants’ treating clinicians, nor will it impact patient care.

The mobile application itself has three modules, which will be described in detail below.

1. Data collection (manual, interactive and passive).
2. Mum-to-be Mental Wellbeing Course.
3. Data visualisation.

#### Data collection

The main aim of the mobile application is to act as a data collection device, which will collect information across a variety of modalities in order to explore which data types are most useful for predicting AD risk and trajectory. The participants will use the app according to their preferences, although notifications will be sent to remind them of specific activities.

##### Manual data entry

With manual data entry, users will be asked to complete standardised mental health questionnaires every fortnight throughout their pregnancy, as detailed in table 1. These measures were chosen to reflect traditional methods of mental health assessment: the Edinburgh Postnatal Depression Scale (for measurement of perinatal-specific depression symptoms),^26^ the Patient Health Questionnaire^27^ (for measurement of general depression symptoms) and the Generalised Anxiety Disorder Assessment^28^ (for measurement of general anxiety symptoms).

##### Interaction with system

The user will directly interact with the system to provide data which can be interpreted for use in mental health prediction. Users will be given access to cognitive tasks two times during their pregnancy, which have been selected to measure domains known to be impacted by depressive symptoms:^29^ spatial memory, mathematical operations and verbal fluency. They will also complete the Stroop test, to measure negative attentional bias, also known to be associated with depressive symptoms.^30^ In addition, it has been shown that perinatal depression can impact a person’s ability to recognise emotion from facial cues.^31^ Therefore, the application includes two emotion recognition tasks^32^,^36^ in which participants will be shown adult faces from the Vienna Emotion Recognition Task database, and infant faces from the Tromso Infant Faces Database,^37^ and asked to identify the emotion shown on the face, chosen from a list.

In addition to the cognitive and emotion recognition tasks which will be made available at specific time points during gestation, users will have access to a number of activities which they can complete at leisure. These include a ‘How was your day’ activity, where users can make verbal answers, and as an optional extra, make a short video recording of their face. These audio and video recordings will subsequently be used to extract acoustic features of speech and video features related to facial affect and expression.

Users will also have access to a ‘Reflections’ exercise, in which they will be able to type short text answers to miscellaneous questions such as ‘If you could meet any historical figure, who would it be and why?’. The typed keys will be collected by keyboard interaction data, as well as the underlying movement of the phone acquired by accelerometer sensors in the phone.

Moreover, users will have access to a mood and events diary, in which they can keep track of their mood as much as they please and identify factors and events which might have contributed to their current mood. They are also able to answer two sleep questions to monitor their quality and duration of sleep. Both mood and events and the sleep diary will be visualised for the user, to aid with their self-monitoring.

Finally, users will have the possibility to report their weight and blood pressure whenever they feel like doing it and up to once a day. The reported data will be displayed to help users monitor themselves during the pregnancy.

##### Passive monitoring

The HappyMums application will ask users for permission to access in-built smartphone sensors, in order to gather data pertinent to physical activity, sociability and phone usage. A detailed description of each data type can be found in table 2.

**Table 2:** Description of data types collected by passive monitoring in the HappyMums application

Passive data type	Description
Pedometer	As a measure of physical activity, number of steps per hour and day will be counted.
GPS	As a measure of participant’s mobility, GPS acquisition in the HappyMums app will be done by adding a random delta of distance (from 10 km to 100 km) to the raw GPS position of the device. The delta will be generated directly on the device, so it will not be known by the backend service and, in this way, guaranteeing anonymisation. Anonymised GPS data will be stored and used to extract different features describing mobility habits, like: number of places visited, time spent in each place, distance travelled and entropy (measurement of uncertainty of data that in behavioural analysis may describe dispersion or clustering of data in a geographical region, ie, high mobility vs limited one).
Accelerometer	As a measure of sleep and activity, this will collect information on phone movement, including shaking, tilting, swinging and rotating.
Light sensor	As a proxy measure for sleep, the app will collect data on the ambient light of the room in which the phone is located.
Screen status	To give information on sleep and phone usage, the app will collect data on when the phone is locked, unlocked and in use.
Call logs	As a measure of sociability, the app will collect information on call frequency, duration and number of missed calls. This will not include specific contact names or phone numbers.
App usage	As a second measure of sociability, the app will be capable of measuring usage of types of apps, for example, time spent using social media applications. The app will not collect the name/reference of the specific apps used.

GPS, Global Positioning System.

##### Mum-to-be Mental Wellbeing course

Users of the application will have access to a library of information and resources, the ‘Mum-to-be Wellbeing course’, which contains information on 21 various topics related to the perinatal period (eg, ‘All about mood: let’s talk about depression in the perinatal period’, ‘Let’s relax: how to relax the body’, ‘The baby: Fetal movements and bonding with your baby’) and has been curated by perinatal experts from the consortium. Further details about the chapters are in online supplemental material. The material is split into content for each of the three trimesters and will be made available to users as they enter their relevant trimester. The main purpose of this course within the HappyMums app is to increase the motivation to use the app and provide relevant verified information about pregnancy and mental health to pregnant women.

##### Data visualisation

The mobile application will provide a space for the visualisation of some data types, for the user to track their own data. This will include information like time spent active during the day and sleep quality from the questions, as well as phone usage (calls, apps categories) or mobility (ie, deltas in GPS data).

### Risk factor assessment

The HappyMums app study aims to build a holistic picture of individualised risk for depressive symptoms during pregnancy, and therefore includes the collection of information pertinent to risk factors identified in the literature. These will be used in exploratory analyses to determine risk factors which are salient in our understanding and interpretation of the application-derived data.

As described above, a list of risk factors will be used as inclusion criteria, to which prospective participants will be asked to select all those which apply. After enrolment, additional details about particular risk factors of interest will be collected from all participants: adult attachment (Adult Attachment Questionnaire^38^), stressful life events (List of Threatening Experiences^39^) and intimate partner violence (Composite Abuse Scale^40^). Additionally, information will be collected on demographic and obstetrical risk factors such as prior history of pregnancy loss, fertility issues, race, ethnicity and socioeconomic status.

In order to improve accessibility to the study and ensure that research burden is not a prohibitive factor for prospective users who may not be able to commit as much time to the study, some risk factor measures are only to be administered in the opt-in comprehensive subset of the cohort. These include couple satisfaction (Couple Satisfaction Index^41^), childhood trauma (Childhood Trauma Questionnaire^42^), perceived social support (Multidimensional Scale of Perceived Social Support^43^), antenatal attachment (Maternal Antenatal Attachment Scale^44^) and perceived stress (Perceived Stress Scale^45^).

### Biological sample collection

An optional component of the study involves the collection of biological samples, with a particular emphasis on participation from those in the comprehensive cohort. Samples will include blood and saliva, collected at 25 weeks of gestation and again at 2 months postpartum. Participants may choose to provide one or both sample types and will also be invited to consent to the collection of saliva samples from their infants at 2 months postpartum.

Blood samples are ideally scheduled for collection between 12:00 and 15:00 to minimise the effects of diurnal variation on analyte levels. However, samples collected outside this time frame will still be included, and all collection times will be recorded. A trained phlebotomist will obtain two serum tubes, two EDTA tubes and two PaxGene tubes from each participant. Samples will be processed and stored at −80°C for subsequent analysis of serum circulating biomarkers related to stress and pregnancy, including hormones and inflammatory cytokines, as well as for DNA extraction.

At both 25 weeks gestation and 2 months postpartum, participants will be sent the necessary equipment to complete saliva collection at home. Participants will be asked to collect saliva throughout the day to assess diurnal cortisol secretion and the cortisol awakening response, due to evidence of disrupted hypothalamic pituitary adrenal (HPA) axis function in women suffering with AD.^46^ Salivary sampling is often favoured over blood collection for measuring cortisol levels due to its simplicity, low cost and non-invasive nature. Moreover, studies examining HPA axis function have reported a strong correlation between salivary and plasma cortisol concentrations.^47^ Samples will be taken at awakening, at 15, 30 and 60 min post awakening, at midday and at 12:00. Sample collection will involve holding a synthetic swab (Starstedt, Leicester, UK) in the mouth for 2 min. These samples will be posted back to researchers, where they will be frozen at −20°C. For analysis of salivary cortisol concentrations, samples will be thawed, centrifuged at 3000 rpm for 15 min and analysed using a commercially available high sensitivity salivary cortisol enzyme immunoassay kit (Salimetrics).

Where specific consent is given, will also be asked to provide an additional saliva sample for DNA analysis (ORAgene, DNA genotek).

### Assessment of mother–infant interaction

Mother–infant interaction will be assessed using the CARE-Index, a reliable and valid coding method used in clinical and research contexts from birth to 15 months. Video interactions will be filmed, and mothers will be instructed to play with their babies as they normally would. Interaction will be assessed across the following dimensions: maternal behaviour, infant behaviour and dyadic synchrony.^23 48^

### Patient and public involvement

The HappyMums consortium has been designed to include the voices of patient representative groups in all aspects of design and implementation. As such, we are grateful to have worked with Tommy’s, a UK-based charity, and the Italian Marcé Society for Perinatal Mental Health, who have provided their own perspective, as well as that of those they work with, at multiple stages in this development process. Surveys were also distributed to potential users (both patients and clinicians) of the app, to gain early feedback during its creation.

### Data analyses

Given the multinational nature of the study, data analyses will be conducted at site-specific and study-wide levels. To address the aim of testing the usage of the app in the at-risk pregnant population, sites will specifically examine their usage data at a site-specific level, before being appraised study-wide. This will be conducted first by developing a set of descriptive analyses to address the primary objective by profiling the proportion of participants who engaged weekly with the mobile application and examining demographic and psychosocial characteristics of active and non-active users, including the antenatal trajectories of depressive symptoms as measured by the EPDS.

Following data completion, study data from all sites will be integrated within a secure federated learning framework to train and evaluate predictive models for maternal depression. This decentralised approach enables sensitive data to remain at local sites while allowing cross-site model training, preserving participant privacy and ensuring diversity in the learnt patterns. Predictive modelling will focus on classifying the risk of AD, as measured by EPDS (Edinburgh Postnatal Depression Scale) scores at baseline, third trimester and postpartum follow-up, with key outcomes defined as binary variables (eg, EPDS ≥13) and continuous trajectories of depressive symptoms over time.

Three supervised machine learning models will be evaluated: Random Forest, Support Vector Machines and logistic regression. These models will be trained on a combination of app-derived and traditional data sources. Input features will be grouped into distinct domains: (1) passive smartphone-derived data (eg, mobility patterns, phone usage, sleep-wake cycles); (2) active self-report data (eg, mood ratings, cognitive performance from app-based tasks); (3) psychosocial and clinical history data (eg, social support, previous mental health diagnoses, adverse life events); and (4) demographic and obstetrical information (eg, age, parity, gestational age, education level).

Model performance will be evaluated using cross-validation techniques within the federated environment. Feature importance will be assessed using internal metrics (eg, Gini importance in Random Forests), and model-agnostic interpretability will be explored via SHAP (SHapley Additive exPlanations) analysis. This will help identify the most influential individual predictors and feature groups contributing to AD risk, aiding interpretability and translational relevance for clinical stakeholders. In doing so, the study aims to provide interpretable, generalisable and privacy-preserving models for early identification and personalised risk assessment of maternal depression.

We acknowledge that there are certain limitations and key risks of this protocol which should be taken into consideration. In terms of data quality and cross-country implementation, we recognise that there is a risk that different languages and cultural contexts could lead to heterogeneity in data collected. To mitigate this, active data collected is in the form of standardised self-report questionnaires which have validated translations. In addition, tasks on the app do not require language input, such as typing speed and voice recognition. Furthermore, researchers on the project have undergone extensive training at all sites to reach reliability in data collection, both online and face-to-face at consortium meetings. As with any study involving the use of technology, we have anticipated technical challenges which could potentially arise, due to different operating systems, phone models and software updates. To mitigate this, the app has been extensively tested by team members at all sites and rounds of feedback have been provided to developers, Ab.acus. The app developers have created in-depth user manuals which will be shared with participants and researchers, to use for troubleshooting, if technical challenges arise. Researchers have trained in guiding participants through any technical errors to ensure smooth usage of the app. As this is a multicentre clinical study, we understand that there might be concern about data sharing and regulations. To mitigate this, a Federated Analysis environment will be developed by the University of Barcelona, whereby models will be trained without data leaving local servers. Furthermore, a Joint Controllership Agreement and Consortium Agreement has been executed by all consortium partners and data collecting sites to ensure ethical use of data. We acknowledge that participants will be completing a battery of self-report measures, which can be burdensome. To mitigate this, a small subgroup, that is, the comprehensive/validation cohort will undergo a more in-depth assessment and will be compensated for their time accordingly. Finally, a current limitation of this methodology is that it excludes individuals who lack access to an appropriate smartphone, who may be part of a deprived demographic group and experience many barriers to care.

### Ethics and dissemination

This study has been registered on ClinicalTrials.gov (NCT06578845) and received approval from ethics committees at each research site. At King’s College London, the study was reviewed by the East of England—Essex Research Ethics Committee and granted favourable opinion (REC reference 24/EE/0129).

The Ethics Committee of the Catholic University of Croatia approved the study in Croatia (Class: 641-03/24-03/003; No: 498-15-06-24-002; Ethics Committee of the Helsinki and Uusimaa Hospital District in Finland (HUS/6890/2024).

The results from this study will be disseminated via publication in peer-reviewed journals and presented at subject-specific conferences. Participants will also be notified of publication of results and provided with a lay summary if desired.

Most recruiting sites have created specific social media accounts to assist with recruitment and dissemination. Social media posts created by Tommy’s are routinely translated and posted on each local site’s account. Furthermore, a website specific to the study has been created.

## Supplementary material

10.1136/bmjopen-2025-106978 online supplemental file 1

10.1136/bmjopen-2025-106978 online supplemental file 2
