# Supplementary material for: HappyMums mobile application study protocol: use of a smartphone application to gather data predictive of antenatal depression
Source: BMJ Open. 2026 Feb 4;16(2):e106978. doi: 10.1136/bmjopen-2025-106978 (PMC12878465; doi:10.1136/bmjopen-2025-106978)
Supplement: online supplemental file 1 [file bmjopen-16-2-s001.docx]

**Screening Questionnaire**

| 1. Have you ever had 2 weeks or more when you felt particularly worried, sad, hopeless or depressed? | YES | NO |  |  |  |  |
| --- | --- | --- | --- | --- | --- | --- |
| 1. Do you have any other history of mental health problems e.g. depression, anxiety, premenstrual dysphoric disorder, eating disorders, psychosis, bipolar disorder, schizophrenia, PTSD | YES | NO |  |  |  |  |
| 1. When you were growing up, did you feel your primary caregiver was emotionally supportive of you? (If you had no primary care figure, circle 6) | 1  very much | 2  ---- | 3  somewhat | 4  ---- | 5  not at all | 6 |
| 1. Is your relationship with your partner an emotionally supportive one? (If you have no partner, circle 6) | 1  very much | 2  ----- | 3  somewhat | 4  ---- | 5  not at all | 6 |
| 1. Have you had any stresses, changes or losses in the last 12 months? (e.g. separation, domestic violence, unemployment, bereavement) | YES | NO |  |  |  |  |
| 1. Would you generally consider yourself a worrier? | 1  very much | 2  ----- | 3  somewhat | 4  ---- | 5  not at all |  |
| 1. In general, do you become upset if you do not have order in your life? (e.g. regular time table, tidy house) | 1  very much | 2  ----- | 3  somewhat | 4  ---- | 5  not at all |  |
| 1. Do you feel you have people you can depend on for support with your baby? | 1  very much | 2  ----- | 3  somewhat | 4  ---- | 5  not at all |  |
| 1. Have you ever been abused? (this could include emotionally, physically or sexually) | YES | NO |  |  |  |  |
| 1. Have you used alcohol, tobacco or any other non-prescription drugs since you found out that you were pregnant? | YES | NO |  |  |  |  |
| 1. Has your sleep worsened since you got pregnant? | 1  very much | 2  ----- | 3  somewhat | 4  ---- | 5  not at all |  |
| 1. Are you experiencing financial stress, or housing or immigration issues? | YES | NO |  |  |  |  |
| 1. Have you experienced any medical conditions or complications related to this pregnancy? (e.g gestational diabetes, hyperemesis, pre-eclampsia) | YES | NO |  |  |  |  |
| 1. Have you experienced fertility issues, the loss of a previous pregnancy, or had a traumatic experience with a previous birth? | YES | NO |  |  |  |  |
| 1. Are you pleased that you are pregnant? | 1  very much | 2  ----- | 3  somewhat | 4  ---- | 5  not at all |  |

Blue highlight = answers equating to eligibility
