# Supplementary material for: HappyMums mobile application study protocol: use of a smartphone application to gather data predictive of antenatal depression
Source: BMJ Open. 2026 Feb 4;16(2):e106978. doi: 10.1136/bmjopen-2025-106978 (PMC12878465; doi:10.1136/bmjopen-2025-106978)
Supplement: online supplemental file 2 [file bmjopen-16-2-s002.docx]

**HappyMums Chapters Topics:**

1. Introduction: what this course is (and what it isn’t!)
2. Let’s begin: your mental health is your baby’s mental health
3. Myths about parenting
4. Circumstances of pregnancy: planned, unplanned, infertility, pregnancy after loss
5. Let’s relax:  how to relax the body
6. Just breathe
7. Fear and anxiety: psychoeducation
8. All about mood: let’s talk about depression in the perinatal period
9. Motherhood and biology: brain, inflammation, hormones
10. The body: weight gain, body image, the history of our body
11. The baby: Fetal movements and bonding with your baby
12. It’s just a thought: something about our way of thinking
13. Assertive communication
14. It’s a question of identity
15. A dip into the past: you as a daughter, your parents and your tough life experiences
16. Delivery: birth plans, the birthing partner, caesarean section
17. A note on sleep
18. Maternal instinct or bonding?
19. Shame, guilt and motherhood
20. Breastfeeding and formula
21. The changing relationships
