# Supplementary material for: HappyMums mobile application study protocol: use of a smartphone application to gather data predictive of antenatal depression
Source: BMJ Open. 2026 Feb 4;16(2):e106978. doi: 10.1136/bmjopen-2025-106978 (PMC12878465; doi:10.1136/bmjopen-2025-106978)
Supplement: Supplementary file 3 [file bmjopen-2025-106978.reviewer_comments.pdf]

PEER REVIEW HISTORY

BMJ Open publishes all reviews undertaken for accepted manuscripts. Reviewers are asked to complete a checklist review form and are provided with free text boxes to elaborate on their assessment. These free text comments are reproduced below.

ARTICLE DETAILS

Title (Provisional)

HappyMums Mobile Application study protocol: use of a smartphone application to gather data predictive of antenatal depression

Authors

Priestley, Kristi; Laijawala, Riddhi; Hazelgrove, Katie; Bind, Rebecca; Rebecchini, Lavinia; Mariani, Nicole; Alford, Sorcha; Kirkpatrick, Madeline; Mancino, Francesca; Kim, Seungyoung; Pushpakanthan, Suvasthiga; Biaggi, Alessandra; Cavaliere, Libera; Di Benedetto, Maria Grazia; Matijaš, Marijana; Žutić, Maja; Brekalo, Maja; Nakić Radoš, Sandra; Żukowska, Katarzyna; Braniecka, Anna; Jackowska, Marta; Bessi, Margherita; Agnoletto, Elena; Melloni , Elisa Maria Teresa; Benedetti, Francesco; Bulgheroni, Maria; La Gamba, Margherita; Martín Isla, Carlos; Izquierdo Morcillo, Cristian; Lekadir, Karim; Salo, Verna; Seikku, Tiina; Räikkönen, Katri; Godara, Malvika; Schneider-Schmid, Ulrike Maria; Entringer, Sonja; Buß, Claudia; de Barra, Deirdre; Woods, Anthony; Dazzan, Paola; Cattaneo, Annamaria; Pariante, Carmine

---

VERSION 1 - REVIEW

**Reviewer**	**1**
**Name**	**Kouroubali, Angelina**
**Affiliation**	**Foundation of Research and Technology Hellas, Institute of Computer Science**
**Date**	**18-Nov-2025**
**COI**	**None**

The study design addresses an important and timely topic. However, while the title identifies this as a feasibility study protocol, the manuscript primarily describes a large multinational data-collection study without clearly distinguishing which components pertain specifically to the feasibility evaluation. It is essential to separate and explicitly define the feasibility aims, outcomes, thresholds for success, and procedures from the broader data-collection protocol.

The limitations of the study are not well articulated, and key risks, particularly those related to data quality, cross-country implementation, participant burden, and technical challenges, are insufficiently discussed. A more comprehensive description of anticipated risks, along

with proposed mitigation strategies, is needed to strengthen the manuscript and ensure transparency.

In the context of related work, additional recent references on digital health applications, especially those relevant to maternal mental health and passive sensing, would improve completeness and situate the study more firmly within the current literature.

Finally, the abstract would benefit from clearer and more balanced reporting of the introduction, methodology, and expected outcomes. As written, it does not fully reflect the scope or methodological complexity of the protocol.

VERSION 1 - AUTHOR RESPONSE

Reviewer's comments:

1. *The study design addresses an important and timely topic. However, while the title identifies this as a feasibility study protocol, the manuscript primarily describes a large multinational data-collection study without clearly distinguishing which components pertain specifically to the feasibility evaluation. It is essential to separate and explicitly define the feasibility aims, outcomes, thresholds for success, and procedures from the broader data-collection protocol.*

We thank the reviewer for considering our study design as important and timely, and we apologise for the lack of clarity. The study has the primary objective to test the 'HappyMums' mobile application in terms of (1) proportion of users who will continue to use the mobile application and engage with its tasks and activities at least weekly from the point at which they are given access to it, until the end of their pregnancy, and (2) usability of the application, measures using the 'Systems Usability Scale' and 'Unified Theory of Acceptance and Use of Technology' questionnaires, administered to users after they cease their use of the application (after giving birth).

As there was no previous data on this application (or indeed on similar purposefully-built apps in the context of depression during pregnancy) we had defined this as a "feasibility" study, but we did not envisage two separate stages or threshold for success, as all the broad data collection was initiated since the beginning of the study. We now understand this was an erroneous use of the term.

To address this comment, we have now removed the term "feasibility" from the title and the description of the study in the paper and the clinical trials registry.

2. *The limitations of the study are not well articulated, and key risks, particularly those related to data quality, cross-country implementation, participant burden, and technical challenges, are insufficiently discussed. A more comprehensive description of anticipated risks, along with proposed mitigation strategies, is needed to strengthen the manuscript and ensure transparency.*

We thank the reviewer for highlighting this important point. To address this, we have included a paragraph on study limitation, which reads as, "We acknowledge that there are certain limitations and key risks of this protocol which should be taken into consideration. In terms of data quality and cross-country implementation, we recognize that there is a risk that different

languages and cultural contexts could lead to heterogeneity in data collected. To mitigate this, active data collected is in the form of standardized self-report questionnaires which have validated translations. In addition, tasks on the app do not require language input, such as typing speed and voice recognition. Furthermore, researchers on the project have undergone extensive training at all sites to reach reliability in data collection, both online and face to face at consortium meetings. As with any study involving the use of technology, we have anticipated technical challenges which could potentially arise, due to different operating systems, phone models, and software updates. To mitigate this, app has been extensively tested by team members at all sites and rounds of feedback have been provided to developers, Ab.acus. The app developers have created in depth user manuals which will be shared with participants and researchers, to use for troubleshooting, if technical challenges arise. Researchers have trained in guiding participants through any technical errors to ensure smooth usage of the app. As this is a multi-center clinical study, we understand that there might be concern about data sharing and regulations. To mitigate this, a Federated Analysis environment will be developed by the University of Barcelona, whereby models will be trained without data leaving local servers. Furthermore, a Joint Controllership Agreement and Consortium has been executed by all consortium partners and data collecting sites to ensure ethical use of data. Furthermore, we acknowledge that participants will be completing a battery of self-report measures, which can be burdensome. To mitigate this, a small subgroup, i.e. the comprehensive/ validation cohort will undergo a more in-depth assessment and will be compensated for their time accordingly. Finally, a current limitation of this methodology is that it excludes individuals who lack access to an appropriate smartphone, who may be part of a deprived demographic group and experience many barriers to care."

3. *In the context of related work, additional recent references on digital health applications, especially those relevant to maternal mental health and passive sensing, would improve completeness and situate the study more firmly within the current literature.*

We thank the reviewer for this suggestion, which will improve completeness and situate the study more firmly within the current literature. We have now added in the following into the introduction:
"Taking this one step further, mHealth technologies have been explored from the perspective of building machine learning models to aid clinical decision making(16). Indeed, evidence from scoping reviews has identified that passive data such as step count, light exposure and speech patterns have been able to predict symptom severity among adult populations with depression. (Amin et al 2025). Within the perinatal context, much of this work has been done in the context of postpartum depression, stemming from electronic health records (17) as well as cohort studies (18). There is, however, a dearth of studies focusing on depression in pregnancy, and machine learning based clinical decision support systems (CDSS) remain underexplored (19). Mobile applications collecting self reported clinical and sociodemographic data have been able to produce highly predictable models of depression in pregnancy (20). Among these, studies integrating passive sensing have been explored in the context of maternal depression, finding that GPS data has indeed been associated with depressive symptomology (21) However, studies looking at integrating passive as well as active data, especially among perinatal populations are limited."

4. *Finally, the abstract would benefit from clearer and more balanced reporting of the introduction, methodology, and expected outcomes. As written, it does not fully reflect the scope or methodological complexity of the protocol.*

Introduction: Mobile health (mHealth) technologies have become increasingly popular for monitoring mental health symptoms and lifestyle behaviours, and are largely reported to be feasible and acceptable to users. However, to date the efficacy of such technologies to improve perinatal mental health outcomes has been mixed.Within the perinatal context, much of this work has been done in the context of postpartum depression, stemming from electronic health records as well as cohort studies. There is, however, a dearth of studies focusing on depression in pregnancy, and machine learning based clinical decision support systems (CDSS) remain underexplored. The HappyMumsss application has been developed to meet this need, and its use across Europe will be tested in this study.

Methods and analysis: A total of 1000 pregnant people currently suffering from, or at risk of, antenatal depression, will be recruited across six countries. All participants will be between 13- and 28-weeks' gestation and will be given access to the new purposefully developed HappyMums mobile application, to use from enrolment until two months postpartum. The application leverages passively collected data from smartphone sensors relating to physical activity and behaviour, as well as requiring active engagement from the user to complete mental health questionnaires and 'game-like' activities. Digital data types will be combined with traditional mental health measurement methods, such as standardised questionnaires and interviews, to develop novel predictive models capable of identifying mental health trajectories in women at risk of developing antenatal depression and to test the app's utility for use as personalised risk prediction and depression identification tool. The primary outcome of this study is to determine what proportion of users will continue to use the mobile application and engage with its tasks and activities at least weekly, while secondary exploratory outcomes include assessing usability of the app, and testing the predictive ability of a novel machine learning based model. These outcomes will, for the first time be assessed by integrating active as well as passive data.

Ethics and dissemination:
Ethical approval has been granted by local research ethics committees in each recruiting centre. At King's College London (leading the clinical study), the study was reviewed by the East of England – Essex Research Ethics Committee and granted favourable opinion (REC reference 24/EE/0129). All other sites collecting participant data have the study approved for local delivery. Findings relating to the primary and secondary outcomes will be submitted for publication in open access, peer reviewed journals, as well as presentations at conferences as symposia or posters. Findings will be made available to a non-specialist audience through open access digital mental health magazines and promotion on social media.

Registration details: This study has been registered on ClinicalTrials.gov (NCT06578845).
